# Clinicopathological and prognostic significance of *DDX41* mutation in myeloid neoplasms: a systematic review and meta-analysis

**DOI:** 10.1007/s00277-025-06278-1

**Published:** 2025-04-21

**Authors:** Liying Miao, Xin Wang, Minghui Yao, Yihao Tao, Yangyang Han

**Affiliations:** 1 Department of Biology, School of Basic Medical Sciences, Xinjiang Medical University, Urumqi, 830017 China; 2 Xinjiang Key Laboratory of Molecular Biology for Endemic Diseases, Xinjiang Medical University, Urumqi, 830017 China

**Keywords:** *DDX41* mutation, Myeloid neoplasms, Clinicopathological features, Prognosis, Meta-analysis

## Abstract

*DDX41* is one of the most frequently altered genes in familial acute myeloid leukemia/myelodysplastic syndrome (AML/MDS). Mutation of *DDX41* has been widely reported in various types of myeloid neoplasms. This systematic review and meta-analysis were conducted to assess the clinical characteristics and relationship between *DDX41* mutations and OS in myeloid neoplasm patients. We thoroughly searched the PubMed, the Cochrane Library, Embase, Web of Science, MEDLINE, and Google Scholar databases. Two reviewers separately reviewed and extracted the data. Twenty studies totaling 9,058 patients have been integrated into the meta-analysis. The extensive pooled analysis showed a significant association between *DDX41* mutations and improved OS (HR 0.70, 95% CI 0.52–0.93, *P* = 0.01). Subgroup analysis confirmed that *DDX41* mutation operated to be a reliable positive indicator of OS when subdivided by different types of myeloid neoplasms. In terms of the clinicopathological value, *DDX41* mutations were significantly correlated with the male sex and older age. AML prevalence, bone marrow, or white blood cell counts did not correlate with any findings. The top three genetic variants were p.M1I, p.D140fs, and p.R525H. Co-mutations in patients with DDX41 mutations most commonly include the following: additional sex combs-like 1 (*ASXL1*), DNA methyltransferase 3 A (*DNMT3A*), tumor protein p53 (*TP53*), ten-eleven translocation 2 (*TET2*) and serine/arginine-rich splicing factor 2 (*SRSF2*). Our results substantiate that *DDX41* mutations were associated with significantly good OS and provide more insight into the clinicopathological characteristics of *DDX41* mutations in individuals with myeloid neoplasms.

## Introduction

Myeloid malignancies are a type of clonal disorders that arise from myeloid stem or precursor cells. These encompass the severe stages, like acute myeloid leukemia (AML), as well as the pre-leukemic phases, which include myeloproliferative neoplasms (MPN), myelodysplastic/myeloproliferative neoplasms (MDS/MPN), and myelodysplastic syndromes (MDS). The advent of next-generation sequencing (NGS) in recent decades has facilitated a more profound comprehension of the molecular pathophysiology of myeloid neoplasms. This has led to the generation of valuable insights pertaining to disease diagnosis, classification, prognosis, and treatment [1–3].

The DEAD/H-box helicase 41 gene (*DDX41*), situated on chromosome 5q35, encodes a protein that functions as a DEAD-box-type RNA helicase. DDX41 comprises four domains: a Coiled coil domain, the two Rec-A like domains of the DEAD-box protein core, RecA1 and RecA2, termed DEAD and HelicC in DDX41 literature, and a Zn-finger domain. Two RecA-like domains contain ten conserved motifs. These ten conserved motifs are involved in ATP binding, ATP hydrolysis, nucleotide binding, and RNA unwinding activities [4]. DDX41 plays a role in RNA conformational changes, including splicing of pre-mRNAs, excision of snoRNA-containing introns, modification of pre-rRNA processing, and interaction with R-loops [5]. 

Heterozygous *DDX41* mutations in the human germline were first detected in multiple MDS and AML families [6]. Through the use of next-generation sequencing technology, 28 different germline *DDX41* variants have been identified in 43 unrelated patients with MDS/AML [7]. Pathogenic mutations in *DDX41* are detected in around 5% of instances of myeloid neoplasms [8]. The *DDX41* mutation was identified as the underlying cause in 80% of myeloid tumors with a genetic background, representing 13% of the total cases [9]. The majority of germline mutations occur in the N-terminus of the protein, predominantly within the DEAD domain, including nonsense, frameshift and missense mutations, which ultimately result in premature termination and truncated proteins, and give rise to protein-level amino acid alterations. Though one of the most prominent predisposition genes for myeloid malignancies is the *DDX41* mutation, the precise implications of *DDX41* mutations in myeloid malignancies remain unclear. For the purpose of precisely characterizing the prognostic significance of *DDX41* mutations in myeloid neoplasm patients and to clarify the associations between *DDX41* mutations and the clinicopathological features of myeloid neoplasms, we conducted a meta-analysis.

## Methods

### Literature registration

The Preferred Reporting Items for Systematic review and Meta-Analysis (PRISMA) criteria were adhered to in the writing of the protocol. The registration number for the protocol in the database PROSPERO is CRD42024508577, and it is accessible via the following link.

(https://www.crd.york.ac.uk/prospero/display_record.php?ID=CRD42024508577)

### Search strategy

The PubMed, the Cochrane Library, Embase, Web of Science, MEDLINE, and Google Scholar databases were used to conduct a systematic literature retrieval of the literature. Retrieval time of included studies is limited until January, 2024 and no language restrictions were applied in the research. The keywords included the following terms: DDX41 or DEAD-box helicase 41; myeloid neoplasms or acute myeloid leukemia or chronic myeloid leukemia or myelodysplastic syndrome. In order to identify any additional eligible publications, the references of key articles were inspected.

### Literature selection criteria

Studies that fulfilled the specified criteria may be incorporated into the meta-analysis: (1)Any type of myeloid malignancies was involved; (2) The relationship between *DDX41* mutations and the resulting clinical outcome; (3) Detailed survival information was provided on patients with *DDX41* mutations to obtain overall survival (OS) data with a hazard ratio (HR) and 95% confidence interval (CI). If the data were not provided, we extracted survival data using the Kaplan–Meier curve. In light of multiple reports pertaining to a single study, our meta-analysis encompassed the publication of the highest quality.

### Data extraction and quality assessment

The data were retrieved by two researchers operating as individual units, and any discrepancies were resolved through discussion among all investigators. The extracted information is as follows: the first author, year, country of the population enrolled, sample types, sample size, percentage of cases with normal karyotype, germline mutation type, subtype of myeloid neoplasms, concomitant mutations, patient’s age and gender demographics, white blood cells, hemoglobin, platelet count, bone marrow blasts, as well as HR and 95% CI of OS.

The methodological quality of the cohort studies included in this analysis was assessed using the Newcastle-Ottawa Quality Assessment Scale (NOS). A NOS score of six or higher signifies that the study is well-conducted and of high quality.

### Statistical analysis

Stata 17.0 software (Stata Corporation, College Station, Texas, USA) and Review Manager (version 5.3, the Cochrane Collaboration, Oxford, UK) were employed to perform the meta-analysis. The relationship between *DDX41* mutations and survival outcomes was evaluated using HR and 95% CI. In the absence of direct offers, the requisite data were extracted from Kaplan–Meier curves via Engauge Digitizer Version 4.1. The resulting rates of survival at particular intervals were then transcribed into the spreadsheet devised by Tierney JF et al. for the calculation of HRs and 95% CIs [10]. Moreover, OR and 95% CI were chosen to estimate the relevance between *DDX41* mutations and clinicopathologic significance. The statistical heterogeneity among the studies was assessed utilizing a chi-squared (χ²) based Q test in conjunction with I² statistics. In the event that heterogeneity is deemed to be significant (*p* < 0.1 or I² > 50%), a random effects model will be employed; if not, a fixed-effects model will be utilized [11]. Publication bias was identified through the application of Egger’s and Begg’s tests, with a P-value of less than 0.05 indicating a significant presence of bias [12]. 

## Results

### Selected studies and characteristic

**Fig. 1:**
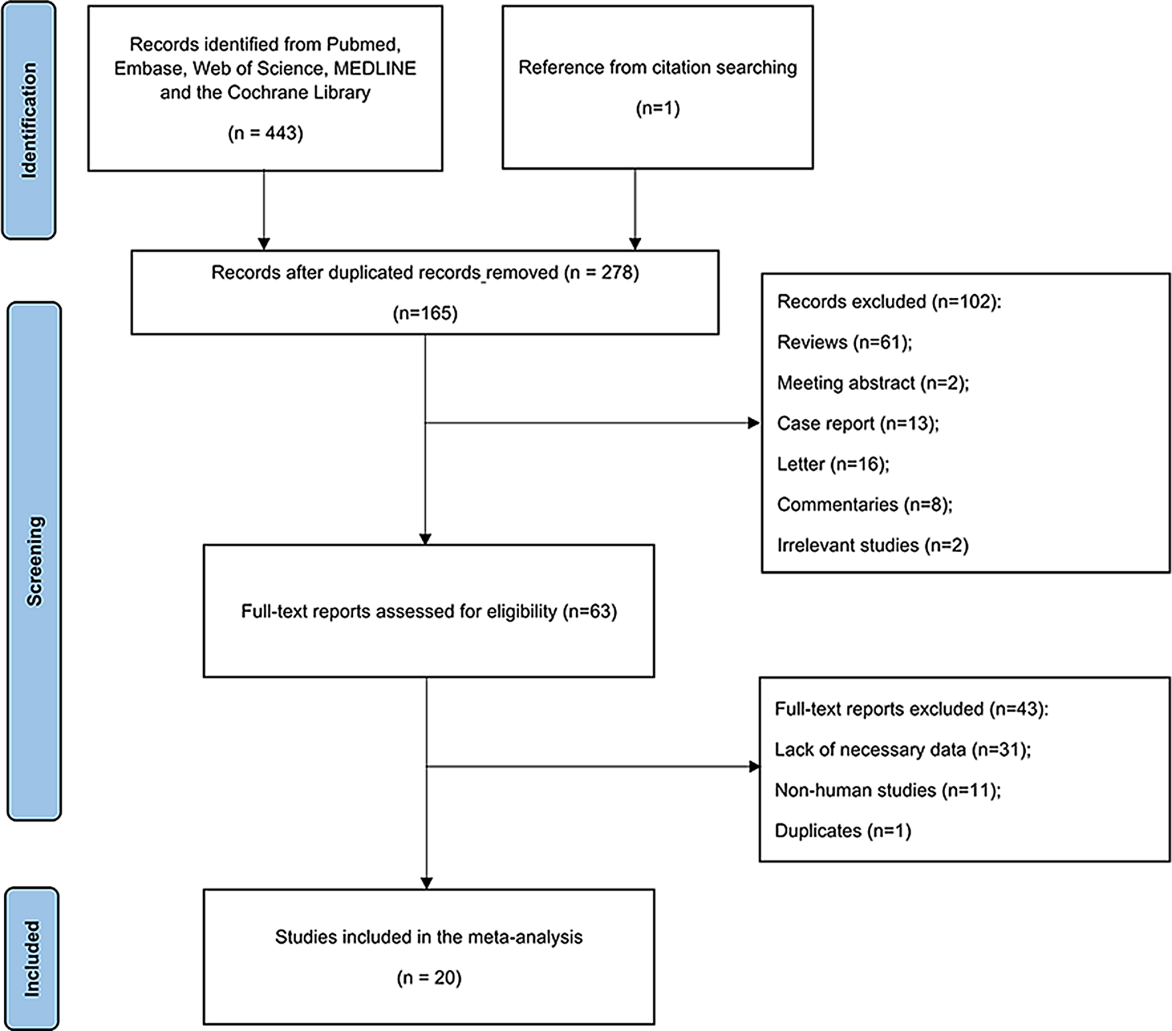
Flow chart for the designated approach

A preliminary literature search yielded a total of 57,097 publications from the PubMed, the Cochrane Library, Embase, Web of Science, MEDLINE, and Google Scholar databases, of which 278 were duplicates. A further study, identified through a review of the references, was deemed eligible for inclusion. A total of 102 articles were eliminated following an in-depth analysis of their titles and abstracts. A further comprehensive examination of the full texts was conducted on the 63 articles, resulting in the identification of 20 publications that ultimately constituted the final meta-analysis [6, 7, 13–30]. The detail of the screening process is demonstrated in Fig. 1. Within the scope of these 20 retrospective studies, a cumulative total of 9,058 patients was examined, with individual sample sizes varying from 2 to 6,336. The range of publication years is given as 2015 to 2023. A summary of the primary characteristics of the research examined is displayed in Table 1. The regions represented in the studies include USA [6, 13, 14, 17, 19–21, 23, 24, 31], China [15, 16], Korea [18], Japan [22], Thailand [26], France [7], Canada [25], Germany [30], Spain [28] and Australia [27]. A variety of myeloid malignancies were observed among the enrolled patients, including MDS, AML, MPN, MDS/MPN, chronic myeloid leukemia (CML), chronic cytopenia of undetermined significance (CCUS), and idiopathic cytopenia of undetermined significance (ICUS)(Table 1) [6, 13, 15, 16, 18, 20–24, 26]. 

**Table 1:** Characteristics of the included studies

Author	Year	Country	DDX41 mutations (*n*)	Gender (M/F)	Age(range)	Subtype	Normal karyotype(%)	NOS score
Quesada et al.[13]	2019	USA	34	26/8	70(45–84)	MDS, AML, MPN, MDS/MPN	58.82	7
Alkhateeb et al.[17]	2021	USA	33	24/9	NR	MDS, AML, MPN, CCUS	NR	8
Badar et al.[20]	2023	USA	107	66/41	NR	MDS, AML, MPN	84.51	6
Bannon et al.[14]	2020	USA	35	28/7	65(48–85)	MDS, AML, MDS/MPN	NR	7
Bataller et al.[21]	2023	USA	151	114/37	69(21–90)	MDS, AML, MDS/MPN	58.60	8
Choi et al.[18]	2020	Korea	28	272/185	66(41–79)	MDS, AML, ICUS	75.00	7
Li et al.[19]	2022	USA	195	109/48	69(7–92)	MDS, AML, MPN	NR	6
Makishima et al.[22]	2023	Japan	346	3940/2396	68(15–94)	MDS, AML, MDS/MPN, MPN	66.90	8
Qu et al.[15]	2020	China	47	44/3	65(33–78)	MDS, AML	98.00	8
S´ebert et al.[7]	2019	France	43	30/13	69(36–88)	MDS, AML, MDS/MPN	NR	8
Zhang et al.[16]	2021	China	14	NR	59(28–78)	AML, MDS/MPN	57.14	7
Goyal et al.[23]	2021	USA	20	10/10	65(45–88)	MDS, AML, MPN, CML, CCUS	68.00	7
Li et al.[24]	2021	USA	28	78/47	68(48–90)	AML	80.00	7
Polprasert et al.[6]	2015	USA	27	645/400	68(44–88)	MDS, AML, MDS/MPN	69.23	7
Tierens et al.[25]	2023	Canada	51	38/13	69(21–91)	MDS, AML, MPN	NR	6
Guijarro et al.[28]	2023	Spain	2	21/26	61,72	AML	NR	6
Jahn et al.[29]	2023	USA	33	NR	NR	AML	NR	6
Maierhofer et al.[30]	2023	Germany	294	NR	NR	MDS, AML, MPN	NR	7
Polprasert et al.[26]	2019	Thailand	6	5/1	67(61–77)	MDS, AML	66.67	7
Singhal et al.[27]	2021	Australia, USA	4	20/10	68(56–84)	MDS, AML	NR	7

MDS: myelodysplastic syndrome; AML: acute myeloid leukemia; MPN: myeloproliferative neoplasm; CML: chronic myeloid leukemia; CCUS: clonal cytopenias of undetermined significance; ICUS: Idiopathic cytopenia of undetermined significance; MDS/MPN: myelodysplastic syndrome/myeloproliferative neoplasms; NR: not reported

### Significance of *DDX41* mutations on prognosis in myeloid neoplasm patients

**Fig. 2:**
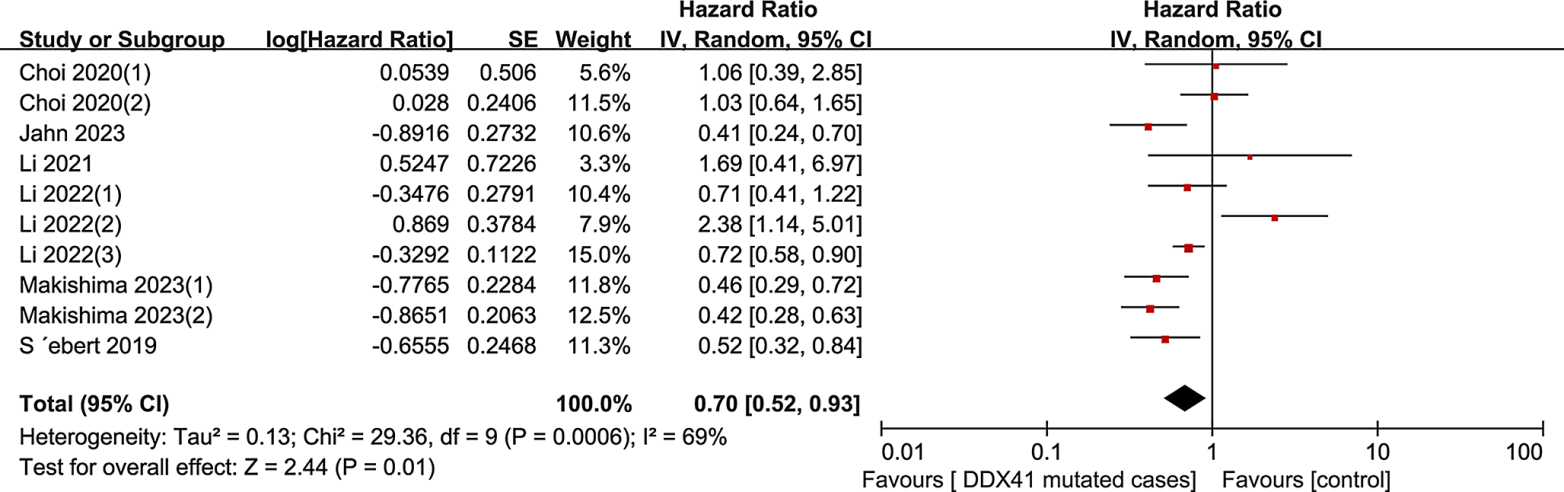
Forest plot for meta-analysis showed the association between myeloid neoplasms patients with *DDX41* mutation and and OS in the entire cohort

**Fig. 3:**
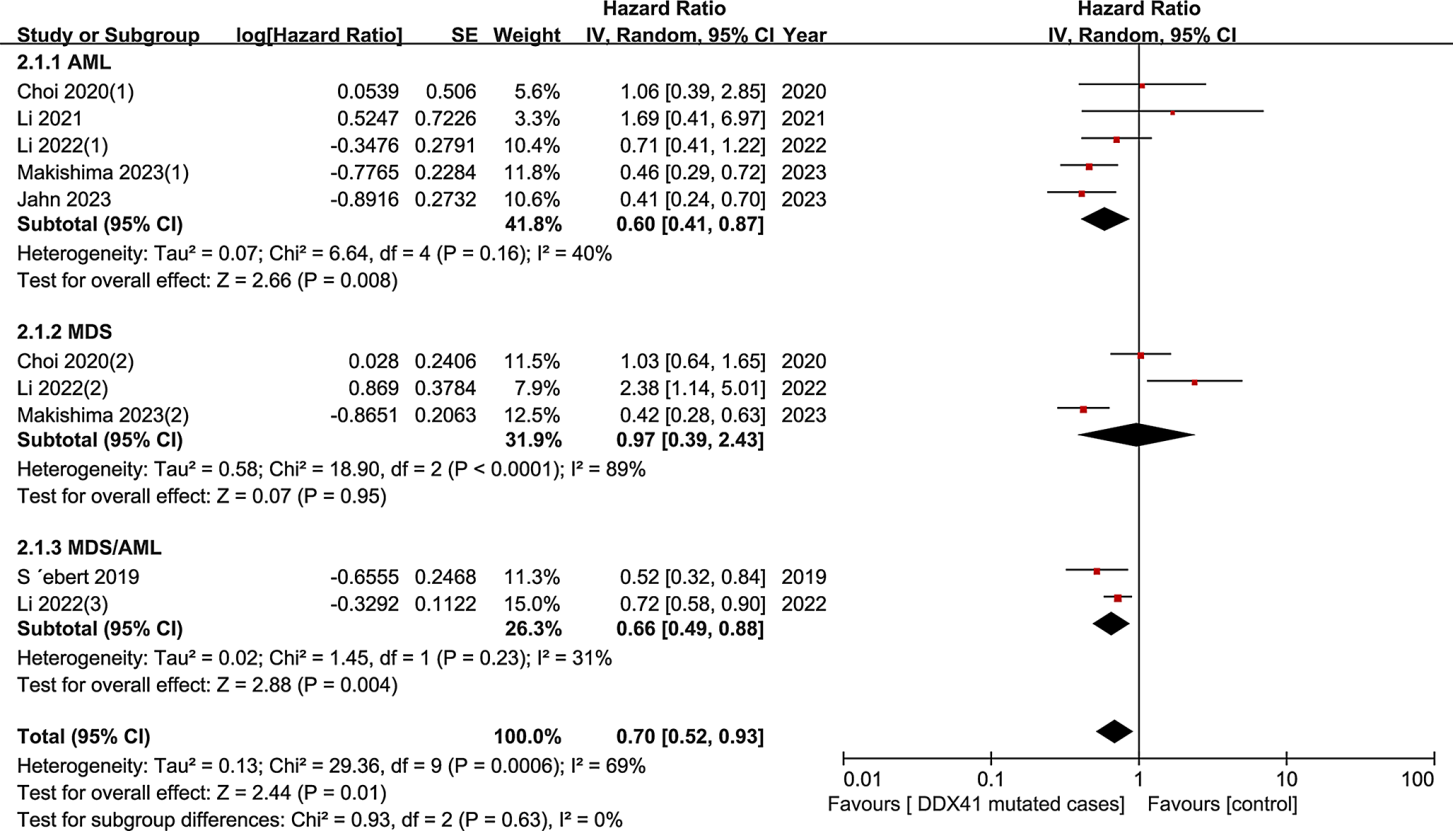
Forest plot of subgroup analysis of OS by myeloid neoplasm type

**Fig. 4:**
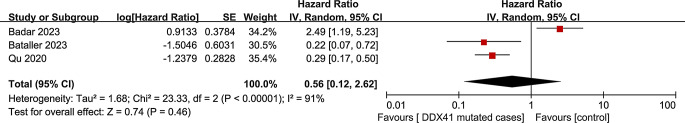
Forest plot for the association of *DDX41* germline and somatic mutations with OS in patients with myeloid neoplasms

We evaluated all eligible studies, the estimated HR for OS was 0.70 (95% CI 0.52–0.93, I² = 69%, *P* = 0.01), indicating that *DDX41* mutation-carrying patients with myeloid neoplasms had a favorable OS compared to *DDX41* wild-type matched controls (Fig. 2). Additionally, to identify the causes of heterogeneity in the OS analysis, a subgroup analysis was used. (Fig. 3). AML patients’ pooled HR was 0.60 (95% CI 0.41–0.87, I² = 40%, *P* = 0.008), according to a subgroup analysis of myeloid neoplasm type, while the HR for patients with MDS/AML was 0.66 (95% CI 0.49–0.88, I² = 31%, *P* = 0.004). With regard to MDS, however, no evidence of a link was discovered (HR 0.97, 95% CI 0.39–2.43, I² = 89%, *P* = 0.95). In addition, three studies provided the data for OS among patients with *DDX41* germline and somatic mutations, though no statistically significant association between them was found (HR 0.56, 95% CI 0.12–2.62, I² = 91%, *P* = 0.46) (Fig. 4).

### Association between DDX41 mutations and clinicopathological characteristics

**Fig. 5:**
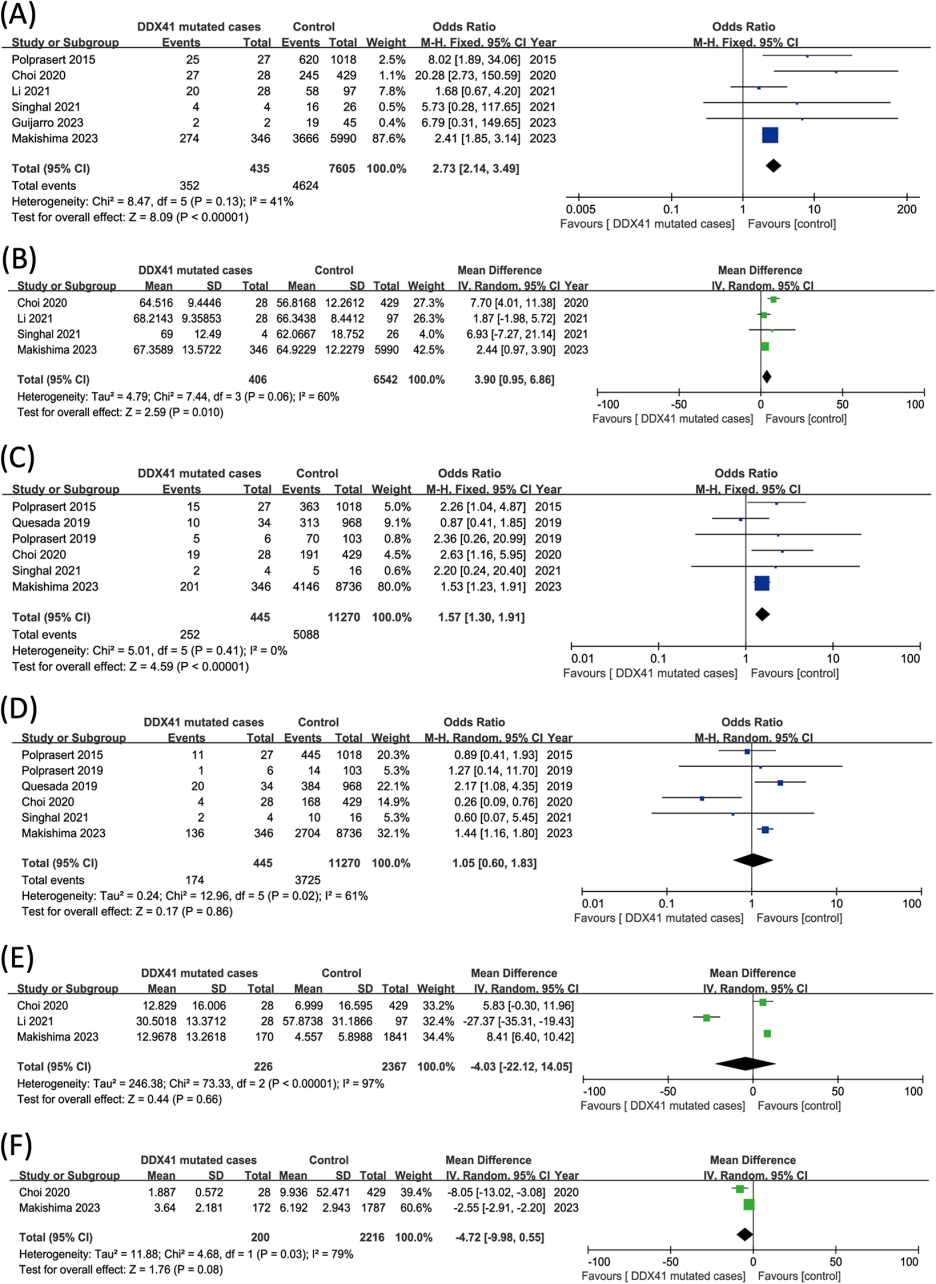
Meta-analysis of *DDX41* mutation association with clinicopathologic. (**A**) Association between *DDX41* mutations and male gender: OR 2.73, 95% CI 2.14–3.49, I² = 41%, *P* < 0.00001. (**B**) Association between *DDX41* mutations and patient age: MD 3.90, 95% CI 0.95–6.86, I² = 60%, *P* = 0.01. (**C**) Association between *DDX41* mutations and prevalence of MDS: OR 1.57, 95% CI 1.30–1.91, I² = 0%, *P* < 0.00001. (**D**) Association between *DDX41* mutations and the prevalence of AML: OR 1.05, 95% CI 0.6–1.83, I² = 61%, *P* = 0.86. (**E**) Association between *DDX41* mutations and blast percentage in bone marrow: MD -4.03, 95% CI -22.12-14.05, I² = 97%, *P* = 0.66. (**F**) Association between *DDX41* mutations and white blood cell count: MD -4.72, 95% CI -9.98-0.55, I² = 60%, *P* = 0.08

Six studies with data on *DDX41* mutation and gender association were identified with a total of 4976 patients for analysis. A noteworthy correlation was discerned between the *DDX41* mutation and male gender (OR 2.73, 95% CI 2.14–3.49, I² = 41%, *P* < 0.00001) (Fig. 5A). Four studies with data on *DDX41* mutation and age of diagnosis of myeloid neoplasms were identified with a total of 6948 patients for analysis. Patients with *DDX41* mutations were diagnosed at an older age compared with those without DDX41 mutations (MD 3.90, 95% CI 0.95–6.86, I² = 60%, *P* = 0.01) (Fig. 5B).

DDX41 mutation related to prevalence of different myeloid neoplasms. The results of five investigations combined showed a strong correlation between prevalence of MDS and the *DDX41* mutation (OR 1.57, 95% CI 1.30–1.91, I² = 0%, *P* < 0.00001), despite no absence of association was demonstrated between *DDX41* mutation and AML (OR 1.05, 95% CI 0.6–1.83, I² = 61%, *P* = 0.86) (Fig. 5C&D). The *DDX41* mutation was not associated with either blast percentage in bone marrow or white blood cells (MD -4.03, 95% CI -22.12-14.05, I² = 97%, *P* = 0.66; MD -4.72, 95% CI -9.98-0.55, I² = 60%, *P* = 0.08) (Fig. 5E&F).

**Fig. 6:**
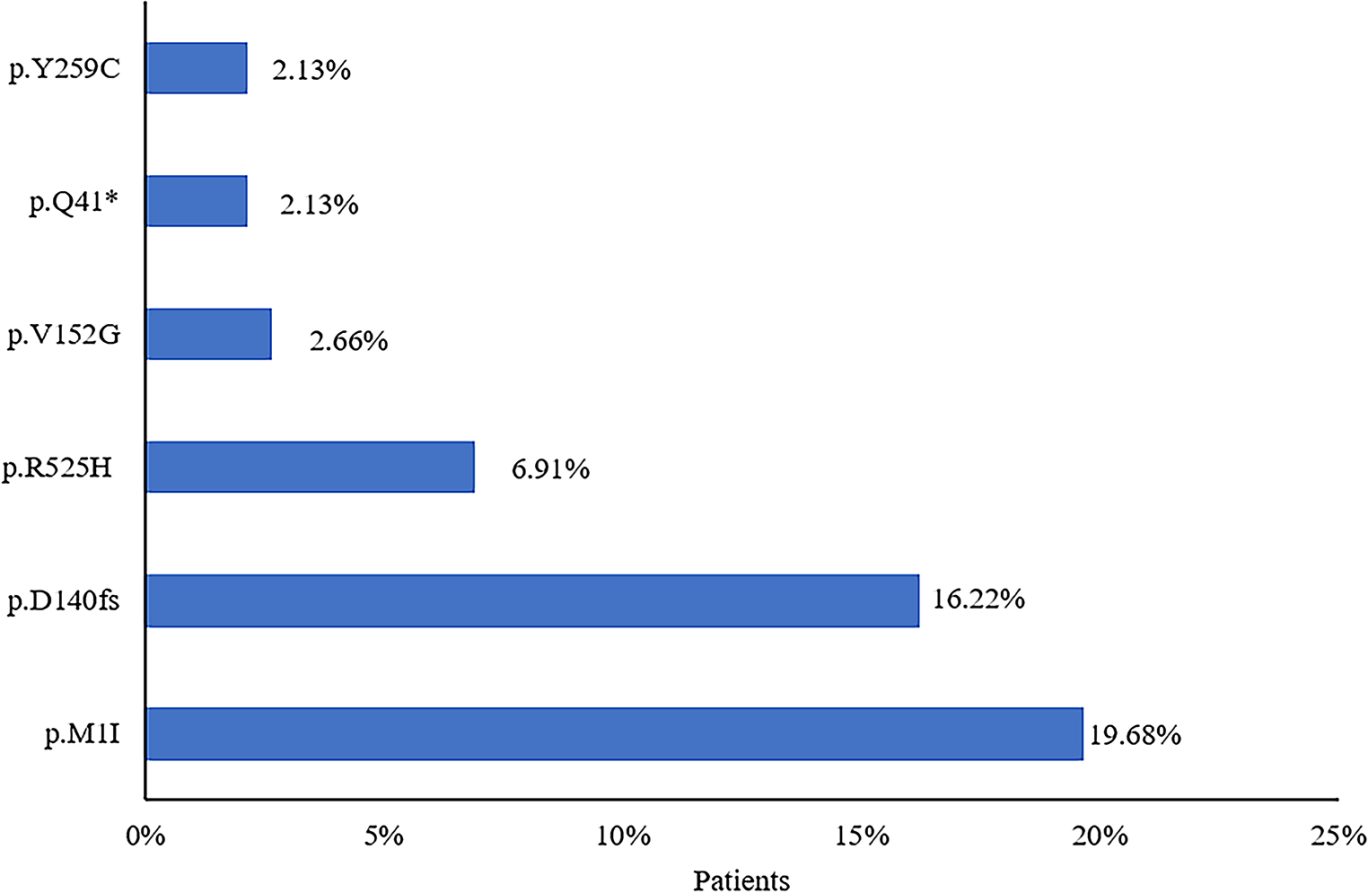
Percentage of patients with germline *DDX41* mutations

Among the 20 publications, 10 articles reported details of germline mutations in 376 patients (Fig. 6). Of the germline *DDX41* mutations, p.M1I (74/376, 19.68%) was most common, followed by p.D140fs (61/376, 16.22%) and p.R525H (26/376, 6.91%). Apart from *DDX41*, the somatic myeloid co-mutations that were most commonly detected were *ASXL1* (172/1077, 15.97%), *DNMT3A* (96/1077, 8.91%), *TP53* (84/1077, 7.8%), *TET2* (60/1077, 5.57%), and *SRSF2* (58/1077, 5.39%).

**Fig. 7:**
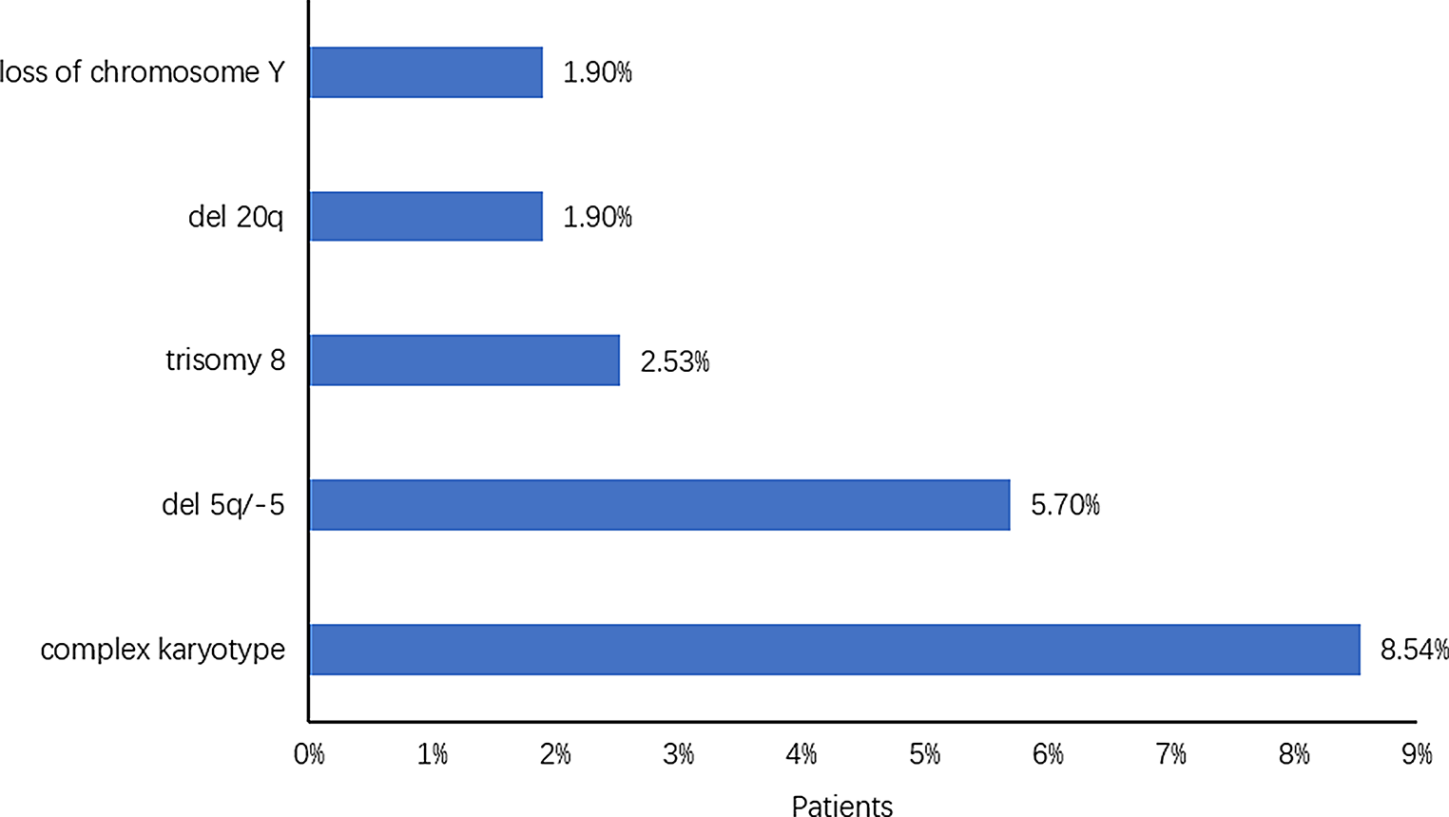
Percentage of patients with abnormal karyotypes

Nine articles reported karyotype information. The frequency of the normal karyotype was higher among *DDX41* mutant patients (Table 1). Complex karyotype (27/316, 8.54%) is the most common, followed by del 5q/-5 (18/316, 5.70%) and trisomy 8 (8/316, 2.53%) (Fig. 7).

### Sensitivity test and publication bias

**Fig. 8:**
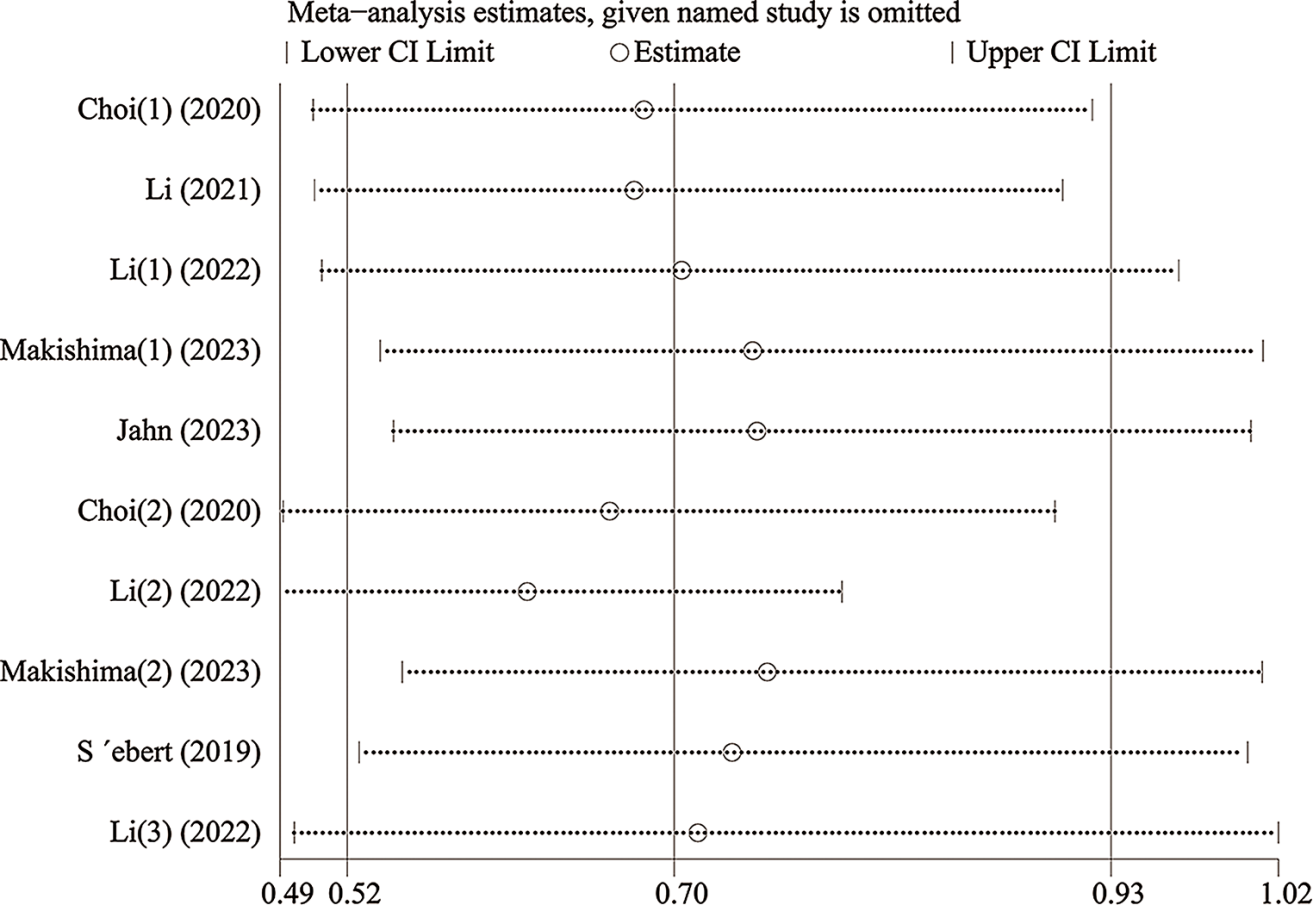
Sensitivity analysis of the meta-analysis of the studies included in the present meta-analysis for OS

**Fig. 9:**
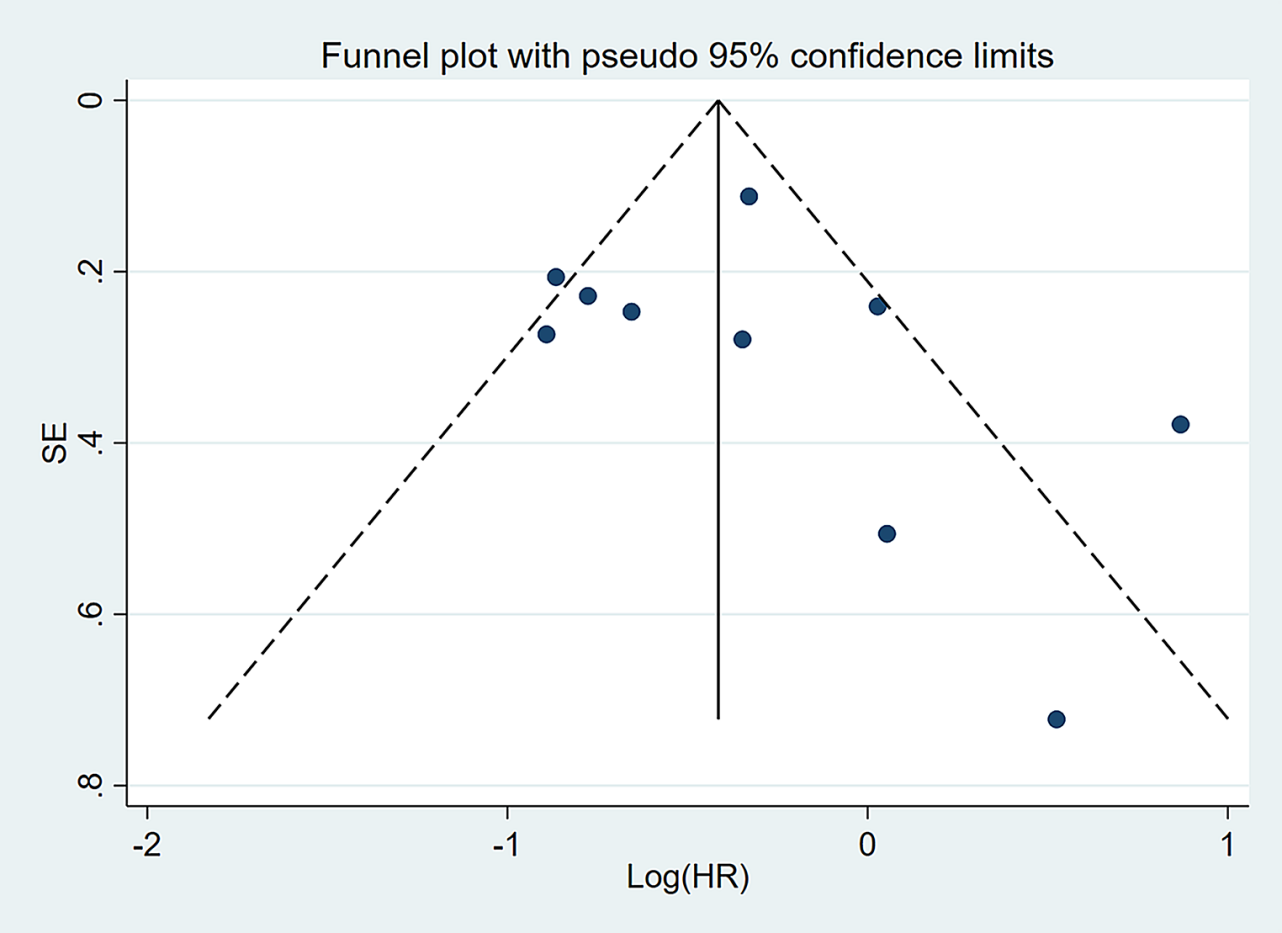
Funnel plot for publication bias in terms of the association of *DDX41* mutation with OS

**Fig. 10:**
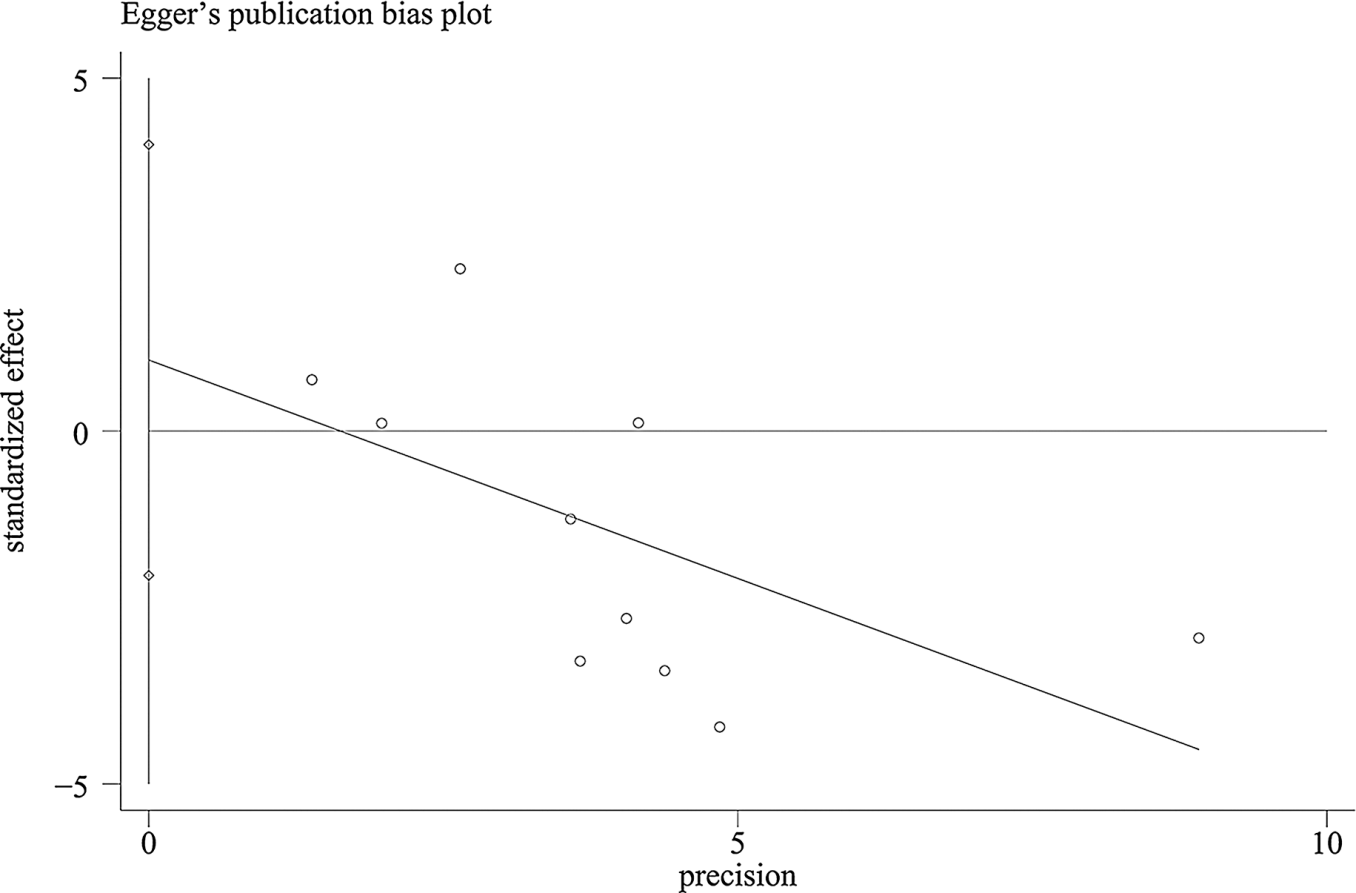
Forest plot of Egger’s test for publication bias of OS

With the aim of evaluating the consistency of the findings and addressing heterogeneity, we performed a sensitivity analysis, whereby each investigation was methodically removed. As shown in Fig. 8, the stepwise exclusion of included studies did not result in any significant differences, suggesting that the individual contributions of each study were inconsequential with respect to the stability of the relationship between *DDX41* mutations and OS in patients diagnosed with myeloid neoplasms. This underscores the robust nature of our outcomes. Utilizing funnel plots, Egger’s, and Begg’s tests, the studies on OS were evaluated for publication bias. As indicated by the funnel plots, there is minimal evidence supporting publication bias (Fig. 9). The Egger’s and Begg’s tests for linear regression also demonstrated the absence of a substantial publication bias (Egger’s *P* = 0.469, Begg’s *P* = 0.210) (Fig. 10).

## Discussion

Virtually every facet of RNA metabolism is carried out by highly conserved enzymes designated as RNA helicases. The families of RNA helicases that are known to exist are DEAD-box, DEAH/RHA, Ski2-like, Upf1-like, and RIG-I [31]. The highly conserved amino acid sequence Asp-Glu-Ala-Asp derived the name of DEAD-box proteins. These enzymes, which exhibit high degrees of conservation among diverse species, participate in a range of RNA-related processes, including ribosome biogenesis, pre-mRNA splicing, transcriptional control, translation, RNA export, and RNA degradation [32]. Two domains resembling DNA recombination and repair protein A (RecA) make up the core of DEAD-box proteins. The DEAD-box protein DDX41 is distinguished by a disordered N-terminal region, a DEAD domain, and a Helicase domain. In patients with myeloid tumors, hereditary and sporadic mutations of *DDX41* have been identified [21, 24]. *DDX41* is a highly mutated gene associated with lengthy latency, severe illness, and a poor prognosis in familial AML/MDS [6]. Despite *DDX41* having been recognized as a tumor-inhibitory gene in myeloid neoplasms, several experiments have assessed the predictive value of *DDX41* mutations in myeloid neoplasms, yet the results remain inconclusive [6, 7]. 

The investigation was based on an analysis of 20 studies encompassing a total of 9,058 patients. Of the 20 retrospective studies, only 6 studies measured survival. As evidenced by the forest plots, meta-analyses have consistently demonstrated that *DDX41* mutations are a powerful prognostic factor for favorable OS. A meta-analysis of existing literature indicates that, when compared with wild-type controls, *DDX41* mutations are positively correlated with OS (HR 0.70, 95% CI 0.52–0.93, *P* = 0.01). Despite the significant heterogeneity (I^2^ = 69%), the sensitivity analysis shows that the meta-analysis’s outcomes were consistent and trustworthy. A comprehensive analysis was undertaken in order to elucidate the association between *DDX41* mutations and myeloid neoplasms prognosis. The data was stratified based on disease type through the undertaking of subgroup analyses. The findings indicated a notable relationship between OS and the AML and MDS/AML cohorts, with pooled HR of 0.60 (95% CI: 0.41–0.87; I^2^ = 40%) and 0.66 (95% CI: 0.49–0.88; I^2^ = 31%), respectively. On the other hand, among MDS patients, nothing indicated a statistically significant link (HR 0.97, 95% CI 0.39–2.43, I² = 89%, *P* = 0.95). Nevertheless, there were only two studies included in the MDS/AML subgroup, and more research is necessary to determine the precise function of mutation detection in OS. It is important to highlight that no statistically significant correlation was identified in patients exhibiting *DDX41* germline and somatic mutations (HR 0.56, 95% CI 0.12–2.62, I² = 91%, *P* = 0.46).

The *DDX41* mutation appears to be connected to male sex, according to our meta-analysis (OR 2.73, 95% CI 2.14–3.49, I² = 41%, *P* < 0.00001). Additionally, our analysis demonstrated a significant association between *DDX41* mutations and older age (MD 3.90, 95% CI 0.95–6.86, I² = 60%, *P* = 0.01), albeit with considerable heterogeneity, underscoring the need for further validation in larger cohorts. The study showed that *DDX41* mutations are strongly linked to MDS prevalence (OR 1.57, 95% CI 1.30–1.91, I² = 0%, *P* < 0.00001), whereas no statistically significant correlation between *DDX41* gene mutation and AML prevalence. This discrepancy may be attributed to the distinct molecular mechanisms underlying MDS and AML, *DDX41* mutations may play a more prominent role in the early stages of myeloid malignancies, such as MDS, rather than in the more genetically complex AML. However, the lack of association with AML prevalence should be interpreted with caution, as it may also reflect the underrepresentation of other myeloid phenotypes, such as MPN, CCUS, and MDS/MPN, in our analysis. Future studies with larger and more diverse cohorts are needed to explore the potential role of *DDX41* mutations in these phenotypes. In addition, no significant effect was found on AML prevalence, bone marrow characteristics, or white blood cell count.

Another prominent feature is that the frequency of normal karyotype was higher in *DDX41* mutation patients. Normal karyotype is associated with favorable prognosis in AML [33], this may partially explain the longer survival observed in patients with *DDX41* mutations. While among abnormal karyotypes, the most prevalent is the complex karyotype, which is linked to poor prognosis in both AML and MDS [34]. In the current analysis, p.M1I, p.D140fs and p.R525H are the most common germline *DDX41* mutations, which have multiple functions in the pathophysiology of a specific category of myeloid neoplasms [35, 36]. Numerous oncogenic mutations have been shown to be prevalent in myeloid neoplasms by genomic profiling investigations. We have included the most often reported co-mutations in patients who have myeloid neoplasms accompanied by *DDX41* mutations. *ASXL1*, *DNMT3A*, and *TP53* were the three most often found co-mutations. It is presently unknown how precisely these co-mutations in *DDX41*-mutated myeloid malignancies work. Nonetheless, a number of investigations have suggested that TP53-mutated *DDX41* mutations may be able to avoid triggering the DNA damage response system, cell cycle arrest, and death [37, 38]. As a result, the ability to survive and undergo more clonal selection may facilitate malignant transformation.

Although this meta-analysis methodically compiles data emphasizing the critical function of *DDX41* mutation as a prognostic factor in myeloid neoplasms, it is imperative to recognize its inherent limitations. Firstly, instead of being prospective research, all of the recruited studies were retrospective designs. A further limitation is that even though we reached out to the authors to request information, there was not enough data to provide statistical power to check for a link. Third, the direct calculation of the HR through the use of the analysis of variance may yield a more accurate result than the HR derived from the survival curve.

In conclusion, we found that OS for myeloid neoplasms is significantly impacted by *DDX41* mutations. AML and MDS/AML were found to be substantially correlated with OS by subgroup screening. Moreover, male sex and *DDX41* mutations were correlated. However, in order to draw a stronger conclusion, prospective randomized controlled trials with a greater diversity of myeloid neoplasm types and bigger sample numbers are required.

## Data Availability

No datasets were generated or analysed during the current study.
